# Supplementary material for: Causal Relationship between Aging and Anorexia Nervosa: A White-Matter-Microstructure-Mediated Mendelian Randomization Analysis
Source: Biomedicines. 2024 Aug 16;12(8):1874. doi: 10.3390/biomedicines12081874 (PMC11351342; doi:10.3390/biomedicines12081874)
Supplement: Supplementary file 1 [file biomedicines-12-01874-s001.zip › Supplementary Figure/Figure S4/Singe SNP plot of MR analysis for TL to ubm-b-2060.pdf]

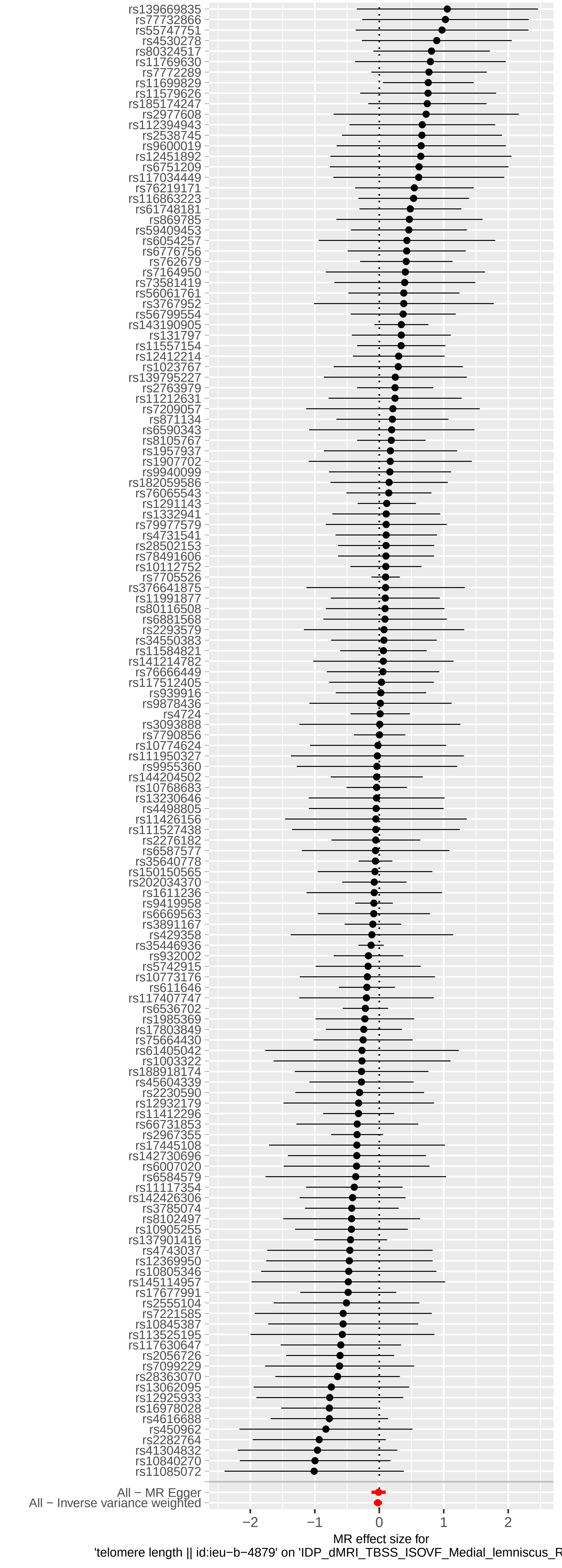

rs139669835
rs77732866
rs55747751
rs4530278
rs80324517
rs11769630
rs7772289
rs11699829
rs11579626
rs185174247
rs2977608
rs112394943
rs2538745
rs9600019
rs12451892
rs6751209
rs117034449
rs76219171
rs116863223
rs61748181
rs869785
rs59409453
rs6054257
rs6776756
rs762679
rs7164950
rs73581419
rs56061761
rs3767952
rs56799554
rs143190905
rs131797
rs11557154
rs12412214
rs1023767
rs139795227
rs2763979
rs11212631
rs7209057
rs871134
rs6590343
rs8105767
rs1957937
rs1907702
rs9940099
rs182059586
rs76065543
rs1291143
rs1332941
rs79977579
rs4731541
rs28502153
rs78491606
rs10112752
rs7705526
rs376641875
rs11991877
rs80116508
rs6881568
rs2293579
rs34550383
rs11584821
rs141214782
rs76666449
rs117512405
rs939916
rs9878436
rs4724
rs3093888
rs7790856
rs10774624
rs111950327
rs9955360
rs144204502
rs10768683
rs13230646
rs4498805
rs11426156
rs111527438
rs2276182
rs6587577
rs35640778
rs150150565
rs202034370
rs1611236
rs9419958
rs6669563
rs3891167
rs429358
rs35446936
rs932002
rs5742915
rs10773176
rs611646
rs117407747
rs6536702
rs1985369
rs17803849
rs75664430
rs61405042
rs1003322
rs188918174
rs45604339
rs2230590
rs12932179
rs11412296
rs66731853
rs2967355
rs17445108
rs142730696
rs6007020
rs6584579
rs11117354
rs142426306
rs3785074
rs8102497
rs10905255
rs137901416
rs4743037
rs12369950
rs10805346
rs145114957
rs17677991
rs2555104
rs7221585
rs10845387
rs113525195
rs117630647
rs2056726
rs7099229
rs28363070
rs13062095
rs12925933
rs16978028
rs4616688
rs450962
rs2282764
rs41304832
rs10840270
rs11085072
All − MR Egger
All − Inverse variance weighted
−2
−1
0
1
2
MR effect size for
'telomere length || id:ieu−b−4879' on 'IDP_dMRI_TBSS_ISOVF_Medial_lemniscus_R
